# Association between selected antimicrobial resistance genes and antimicrobial exposure in Danish pig farms

**DOI:** 10.1038/s41598-017-10092-9

**Published:** 2017-08-29

**Authors:** Anna Camilla Birkegård, Tariq Halasa, Kaare Græsbøll, Julie Clasen, Anders Folkesson, Nils Toft

**Affiliations:** Division for Diagnostics & Scientific Advice, National Veterinary Institute, Technical University of Denmark, Kemitorvet Building 204, 2800 Kgs. Lyngby, Denmark

## Abstract

Bacterial antimicrobial resistance (AMR) in pigs is an important public health concern due to its possible transfer to humans. We aimed at quantifying the relationship between the lifetime exposure of antimicrobials and seven antimicrobial resistance genes in Danish slaughter pig farms. AMR gene levels were quantified by qPCR of total-community DNA in faecal samples obtained from 681 batches of slaughter pigs. The lifetime exposure to antimicrobials was estimated at batch level for the piglet, weaner, and finisher periods individually for the sampled batches. We showed that the effect of antimicrobial exposure on the levels of AMR genes was complex and unique for each individual gene. Several antimicrobial classes had both negative and positive correlations with the AMR genes. From 10–42% of the variation in AMR gene levels could be explained in the final regression models, indicating that antimicrobial exposure is not the only important determinant of the AMR gene levels.

## Introduction

Antimicrobial consumption in pigs is a major contributor to the global antimicrobial consumption in livestock^1^. In Denmark, approximately 66% of the antimicrobials consumed are purchased for use in livestock of which 76% are used in pig production^2^. High levels of antimicrobial resistance (AMR) are therefore expected in Danish pig farms due to the selective pressure of the antimicrobials consumed. Pigs constitute a potential reservoir of AMR that can be transferred to pathogenic bacteria in humans through pork, direct contact with pigs, or release of porcine manure into the environment^3, 4^. The rapid increase of AMR in recent decades has intensified the discussion about the prudent use of antimicrobials, especially in the pig production.

AMR is a natural consequence of the selective pressure of antimicrobials. However, the relationship between antimicrobial exposure and AMR is not easy to quantify^5^. Many AMR-associated genes have other functions not related to AMR when antimicrobial exposure is absent^6^. Epidemiological factors, including the size and age group of the population at risk and contact between farms, further complicate the quantification of the relationship between antimicrobial exposure and AMR. The population size is important as it directly relates to the antimicrobial exposure^5, 7^, and age is important as the composition of the intestinal microflora changes with the age of the pig^8^. On a pig farm, animals are normally housed in groups based on age, so information about the size and age of the population at risk is easily obtainable. Data on the purchase of antimicrobials and information on contacts between farms in Danish pig production are available, making the pig farm an ideal study unit for quantifying the relationship between antimicrobial exposure and AMR.

Previous studies estimating the relationship between AMR and antimicrobial exposure in pig populations have primarily focused on phenotypic resistance in one or few bacterial species^9–15^. This method underestimates the risk of AMR genes present in porcine faeces, as large parts of the gut microbiota cannot be cultured by traditional means^16^. Quantitative Real-Time Polymerase Chain Reaction (qPCR) is a DNA-based method extensively used to monitor gene levels due to its quantitative precision, low contamination risk, high sensitivity and broad dynamic range^17^. With qPCR, it is possible to quantify the levels of AMR genes from total-community DNA, even in complex samples such as porcine faeces^18, 19^.

The factors driving AMR in pig production requires investigation in order to introduce efficient initiatives to reduce the levels. These factors are best studied in environments reflecting real-life practices. The cross-sectional study design is one of the preferred methods of studying the status of a population as it is relatively cheap and thus enables a large sample size.

The objective of this study was to quantify the association between the lifetime exposure of pigs to antimicrobials and the levels of seven AMR genes, *ermB*, *ermF*, *sulI*, *sulII*, *tet*(M), *tet*(O), and *tet*(W) in Danish slaughter pig farms.

## Results

### Population

Faecal samples were obtained from 681 batches of slaughter pig from Danish pig farms. Samples from one batch per farm were included in the study. The samples were collected at five abattoirs that only slaughtered pigs weighing approximately 100–120 kg (five to six months of age^20^). Thus, the influence of age on the AMR level^21^ could be excluded as a bias.

The antimicrobial exposure for the batches was calculated as the average amount of antimicrobials to which pigs in the batch were exposed during their lifetime. The antimicrobial exposure estimates were calculated using information about antimicrobial purchases, farm demographics, and pig movements obtained from national registers (see methods section for details). However, it was not possible to calculate the antimicrobial exposure for 46 batches (7%), due to missing data in the registers. These were excluded from further analysis, resulting in a total of 635 batches of slaughter pigs included in the final analysis. Non-detects for *tet*(M) were found in samples from 35 batches (6%) and for *ermF* in samples from 15 batches (3%). These observations were excluded from the analyses where *ermF* and *tet*(M) were included.

### Descriptive analyses

**Figure 1:**
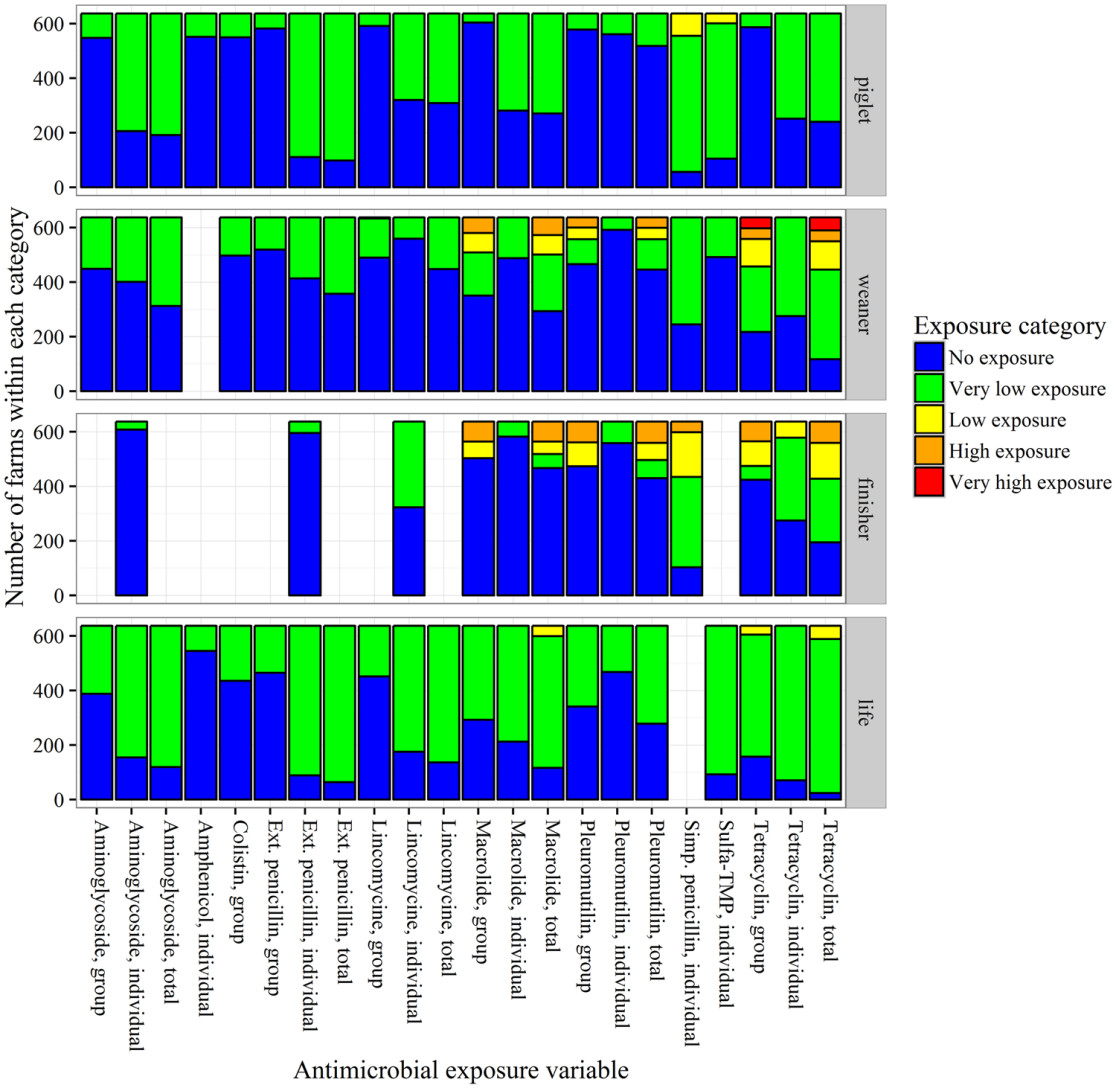
Categories for antimicrobial exposure variables. The number of categories within each antimicrobial exposure variable and the distribution of batches within the categories. Simp.: simple. Ext.: extended. Cephalosporins were given to very few batches and therefore not included.

The antimicrobial exposure was estimated for each batch of pigs as the average animal daily dose for treatment of one kg pig (ADD_kg_) for each of three rearing periods; piglet (birth-7 kg), weaner (7–30 kg), and finisher (30 kg-slaughter) period and as a lifetime total. Due to the heterogeneous pattern of antimicrobial consumption in Danish pig production, antimicrobial variables were categorised using the values given in Table 1. The resulting number of groups per antimicrobial exposure variable can be seen in Fig. 1. Groups were merged if one group represented less than five percent of the batches. Antimicrobial exposure variables were excluded from the analyses when all batches were in the same group, which among others was the case for cephalosporins (all usages), sulfa-TMP as group treatment, and colistin as individual treatment (Fig. 1).

**Table 1:** Average animal daily dose (ADD) per kg pig intervals used to categorise the antimicrobial exposure.

Period	Exposure levels				
	No	Very low	Low	High	Very high
Piglet	0	[0;87]	[87;136]	[136;186]	[186;522]
Weaner	0	[0;52]	[52;100]	[100;167]	[167;2777]
Finisher	0	[0;17]	[17;66]	[66;137]	[137;1367]
Lifetime	0	[0;239]	[239;348]	[348;474]	[474;2900]

The intervals are given by the summary statistics for the total antimicrobial exposure in each rearing period (piglet, weaner, finisher and lifetime) No exposure: a value of zero; Very low exposure > 0–25^th^ percentile; Low exposure (>25^th^ percentile - Median); High exposure (>Median - 75^th^ percentile); Very High exposure (>75^th^ percentile).

### Regression analyses

Regression analyses were made to assess the quantitative relationship between antimicrobial exposure and AMR gene levels. Logistic multivariable regression analyses were made for *ermB* (low/high levels) and for *sulI* and *sulII* (absence/presence). Linear multivariable regression analyses were made for *ermF*, *tet*(M), *tet*(O), and *tet*(W) with the levels presented as log(RQ-value). The effect of the variables in the final regression models can be seen in Supplementary Tables S1–S7 for the *ermB*, *ermF*, *sulI*, *sulII*, *tet*(M), *tet*(O), and *tet*(W) genes, respectively. We showed that batches exposed to high levels of macrolides in the finisher period had 66 times higher odds of having a high level of *ermB* than baseline batches that were not exposed to macrolides in the finisher period (Supplementary Table S1). Furthermore, the RQ value of *ermF* that was increased with 2.5 in batches that were exposed to high levels of macrolides compared to batches that had not been exposed to macrolides in the finisher period (Supplementary Table S2, 2.5 is a value of 0.92 on the log scale). The number of explanatory variables that were significant in the final models ranged from three (the *tet*(O) model, Supplementary Table S6) to eight (the *ermF* and *tet*(W) models, Supplementary Tables S2 and S7). The proportion of the gene variation explained by each model was as follows: *ermB* = 42%; *ermF* = 29%; *sulI* = 10%; *sulII* = 10%; *tet*(M) = 10%; *tet*(O) = 18%; *tet*(W) = 35%. Collinearity was not found between any of the continuous explanatory variables. Furthermore, exposure to amphenicol, colistin, and sulfa-TMP exposure did not correlate with any of the AMR genes.

**Figure 2:**
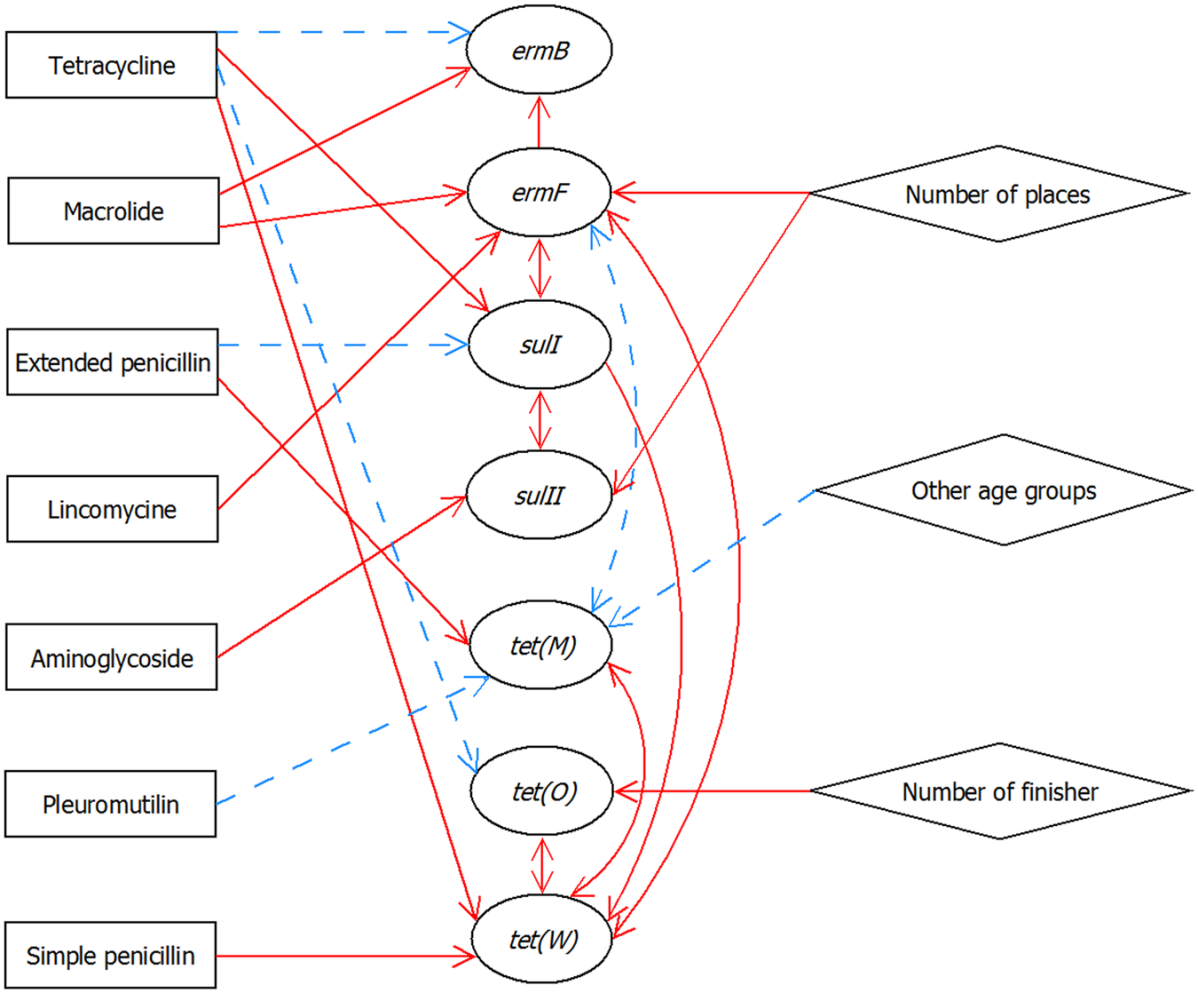
Association between antimicrobial exposure and antimicrobial resistance genes. Map of association showing the effect of antimicrobial exposure variables and other factors on the levels of antimicrobial resistance genes. The figure is a summary of the effect of antimicrobial class and other variables included in the final regression analyses given in Supplementary Tables S1–S7. A dotted blue line indicates a negative correlation and a solid red line indicates a positive correlation.

The complexity of the association between antimicrobial exposure and the seven AMR genes is summarised in Fig. 2. We found 23 positive correlations (Fig. 2, red solid lines) and 8 negative correlations (Fig. 2, blue dotted lines). Exposure to tetracycline was negatively correlated with *ermB*, and *tet*(O), while being positively correlated with *sulII*, and *tet*(W). This mixed correlation with AMR genes was also the found for exposure to extended penicillins and tetracyclines. Exposure to macrolides, simple penicillins, lincomycins, and aminoglycosides was positively correlated with several AMR genes. Exposure to simple penicillins was negatively correlated with *tet*(W).

## Discussion

This study highlights the complexity of the relationship between antimicrobial exposure and AMR genes. The quantitative association of antimicrobial exposure and AMR genes depended on the specific gene as well as the antimicrobial class. While the exposure to tetracycline and extended penicillin was positively correlated with the level of certain AMR genes, it was negatively correlated with the level of others (Fig. 2). Even genes conferring resistance to the same antimicrobial class showed differently correlation patterns with exposure to antimicrobial classes in our study (Supplementary Tables S1–S7).

The association between antimicrobial class and AMR genes encoding resistance against that particular class was expected. We found that exposure to macrolides and lincomycin was positively correlated with *ermB* and *ermF*, and tetracycline exposure was positively correlated with the levels of *tet*(W). The association could be the result of selective pressure by the antimicrobial agent on the bacterial flora increasing the AMR gene levels, or the presence of the AMR gene necessitating a higher dose of the antimicrobial agent in order to treat the bacterial infection. However, tetracycline exposure was not correlated with *tet*(M), nor was sulfa-TMP correlated with the *sul* genes. Perhaps because sulfa-TMP is rarely given to younger pigs (7 kg–100 kg), but is often used to treat sows^2^. Furthermore, we found a negative correlation between exposure to tetracycline and *tet*(O). This could be due to tetracycline concentrations in the pig gut are above the minimal inhibitory concentration that this gene confers. Alternatively, antimicrobial treatment may change the composition of the gut microbiome so that microbes harbouring *tet*(O) decrease in number relatively to the total number of microorganisms in the gut even though the genes confers increased protection to tetracycline. Furthermore, this could be explained by the inclusion of the *tet*(W) gene in the final regression model.

Tetracycline exposure has repeatedly been shown to increase levels of phenotypic tetracycline resistance^10–12, 14, 22, 23^. However, high levels of phenotypic^9, 10, 12, 21^ and genotypic^14, 24, 25^ tetracycline resistance have also been found in pigs that have not been exposed to tetracycline. This inconsistent relationship between antimicrobial exposure and AMR has also been shown for macrolides^24, 26, 27^. This corresponds well to a recent systematic review, where no clear association was identified between antimicrobial exposure and phenotypic AMR^28^, perhaps due to different specific AMR genes driving the phenotypic AMR.

We found that some antimicrobials decrease the prevalence of some genes (e.g. the effect of tetracycline exposure on *ermB* and aminoglycoside exposure on *sulI*, Fig. 2). This might be because a decrease in the use of one antimicrobial class will normally be accompanied by an increase in another^29^. For example, farms using few tetracyclines might use more macrolides. Therefore, the decreasing effect of tetracycline on *ermB* could have been a hidden effect of macrolide use.

To our knowledge, this is the first study to quantify the association between antimicrobial use and AMR in pigs using a lifetime antimicrobial exposure estimate and AMR genes in total-community DNA. Although the relationship between AMR levels and antimicrobial exposure in pigs has been subject to several studies, the present study differs in some important aspects. Previous studies have either: monitored a relatively narrow period in the production cycle^27^; focused on one or few bacterial species^9, 22, 23, 30^; included only few antimicrobials^24^; omitted information on the antimicrobial exposure prior to the study period^26^; or lacked information about antimicrobial exposure^30, 31^. Furthermore, because detailed register data were available at farm level, it was possible to calculate antimicrobial exposure for almost all batches of pigs from which we had samples.

Our findings suggest that AMR genes in pigs at the time of slaughter potentially were affected by antimicrobial exposure during the entire rearing period. This was expected given the relatively short lifespan of a slaughter pig, which is five to six months in Denmark and consistent with what has been previously shown^9, 11, 13^.

Determining the AMR gene levels in the total-community DNA is challenging, since neither the proportion of AMR genes nor the bacteria that harbour the genes are known. However, all seven AMR genes have been shown to occur on mobile elements, and can therefore be transferred from one bacterium to another^32–34^.

We used lifetime antimicrobial exposure at batch level as a proxy for antimicrobial consumption for the pigs. The term ‘exposure’ is deliberately chosen as the exact consumption of the individual pig cannot be established based on register data. In a pig farm, the animals are exposed to antimicrobial residues excreted by treated pigs as well as antimicrobials administered orally or parenteral. Furthermore, pigs excrete AMR genes to the environment in the faecal droppings, which might be indigested by other pigs resulting in spread of AMR genes within a pig pen. Therefore, our hypothesis was that all antimicrobials used at a farm can contribute to the AMR gene levels in the pigs present at the farm.

We also showed that factors other than antimicrobial exposure were associated with the AMR gene levels. For example, *tet*(O) was positively correlated with the number of slaughter pigs present at the farm, which has also been shown for phenotypic tetracycline resistance^10^. In our study, we used the number of farms from which the pigs originated as a proxy for the degree of mixing and transportation. This number was correlated with the levels of *sulII* and *ermF* (Fig. 2, Supplementary Tables S2 and S4). We also found that the levels of AMR genes were correlated with those of other AMR genes, which could be the result of either co- or cross-resistance. This was further supported by the correlations found between exposure to penicillins, simple and extended, was correlated with *sulI*, *tet*(O) and *tet*(W) (Fig. 2).

We were only able to explain 10–42% of the variation in AMR gene levels by factors included in the statistical analyses. A possible reason could be non-antimicrobial risk factors known to affect the AMR levels, but not included in this study due to information not being obtainable from available registers. These include transportation^35^, housing temperature^35^, farm management^23^, and the consumption of metals^36^. Furthermore, the bacterial composition of the porcine gut and feeding strategies also affect the levels of the AMR genes because many bacteria are intrinsically carrying AMR genes^6^.

In conclusion, using lifetime estimations of antimicrobial exposure and levels of AMR genes in total community DNA we quantified the associations between antimicrobial exposure and the level of AMR genes. These associations were found to be more complex than previously described and depended both on the specific AMR genes and antimicrobial classes in question. Furthermore, our results indicate that antimicrobial exposure is not the only important determinant of the AMR gene levels.

## Materials and Methods

### Study design

This paper follows the recommendations to optimise reporting of epidemiological studies on antimicrobial resistance (STROBE-ASM guidelines^37^).

The study design was cross-sectional with a target population of Danish pig farms with conventional production of slaughter pigs. The samples were obtained by a tested and validated method for sampling at abattoirs in order to ensure that the resulting samples were representative of the target population as explained by Birkegård *et al*.^38^. Information about the farms was obtained through national registers after sampling. The sampled pigs had no clinical signs of disease as they were assessed suitable for slaughter, and antimicrobial exposure was a result of treatments for diseases occurring in a normal Danish pig production. Almost all pigs were exposed to antimicrobials in one or more rearing periods. In Denmark, antimicrobial use for growth promoting or prophylaxis is not permitted^39^ and therefor the reported antimicrobial usage are for treatment of diseases or use in metaphylaxis.

The study unit was a batch – defined as a group of pigs slaughtered on the same day and originating from the same farm.

Faecal samples from pigs slaughtered in Denmark were collected in February and March 2015 at five abattoirs. The number of farms to sample was determined by the available resources, and sampled farms were selected randomly, as previously described^38, 40^. In brief, the faecal samples were collected at the slaughter line after removal of the gut from the carcass. A small amount of faecal material was squeezed out of the rectum of the removed gut and into an empty 12.5 mL sampling vial. Five pigs were sampled per farm as this was shown to be sufficient to account for the variation in AMR at farm level^18^. The five faecal samples were pooled resulting in one sample per farm.

The farmers were not informed of the sampling as their cooperative abattoir management gave permission. Therefore, selection bias in terms of willingness to participate in the study can be excluded. However, the sample scheme resulted in a sampling bias towards larger farms^38^.

### Level of antimicrobial resistance

In this study, the levels of seven AMR genes (*ermB*, *ermF*, *sulI*, *sulII*, *tet*(M), tet(O), and *tet*(W)) were quantified. These genes were chosen because an assay had been validated for these specific genes in a previous study^19^. Inclusion criteria for the selected genes were: 1) the use of the antibiotic class in the Danish pig production, 2) the occurrence of the gene in a wide bacterial population and 3) the possibility of designing a qPCR assay for the chosen genes utilizing the same temperature profile^19^.

Based on the pooled samples the AMR levels were quantified, as described by Clasen *et al*.^18^. DNA was extracted using the Maxwell 16 Blood DNA Purification Kit (Promega Corporation, Madison, WI, USA) and DNA concentrations were diluted to 40 ng/µl. Levels of seven AMR genes were quantified using the high-capacity qPCR chip Gene Expression 192 × 24 (Fluidigm Corporation, South San Francisco, CA, USA) with two technical replicates using 16S as the reference gene, as previously described^38^. The average cycle of quantification (C_q_) value for the two technical replicates was used in the further analyses. C_q_ values above the gene specific limit of quantification were regarded as non-detects. The gene specific limits of detection were 23 (*ermB*, *sulII*, *tet*(O)), 24 (*ermF*, *tet*(W), 16S), 25 (*tet*(M)) and 26 (*sulI*) respectively and efficiencies ranged from 90.9–108.2%^18^. After excluding samples with non-detects, obtained Cq values were corrected for variations in between runs, by the use interplate calibration followed by correction for efficiency of the genes. Relative quantification (RQ) values were calculated from cycle of corrected Cq values with the modified Livak method^41^ (equation (1))

1 $${{\rm{R}}{\rm{Q}}}_{{\rm{p}}{\rm{r}}{\rm{i}}{\rm{m}}{\rm{e}}{\rm{r}}{\rm{s}}{\rm{e}}{\rm{t}}{\rm{X}}}={2}^{-({\rm{C}}{\rm{q}},{\rm{g}}{\rm{e}}{\rm{n}}{\rm{e}}{\rm{o}}{\rm{f}}{\rm{i}}{\rm{n}}{\rm{t}}{\rm{e}}{\rm{r}}{\rm{e}}{\rm{s}}{\rm{t}}-{\rm{C}}{\rm{q}},{\rm{r}}{\rm{e}}{\rm{f}}{\rm{e}}{\rm{r}}{\rm{e}}{\rm{n}}{\rm{c}}{\rm{e}}{\rm{g}}{\rm{e}}{\rm{n}}{\rm{e}})}$$

Due to the large number of non-detects among the samples for *sulI* and *sulII*, these genes were dichotomised as present or absent^38^. The distribution of RQ values for the *ermB* genes was skewed^38^, so the levels of RQ values were therefore classified as either low (below the 75^th^ percentile) or high (above the 75^th^ percentile).

### Level of antimicrobial exposure

The lifetime exposure to antimicrobials was calculated as the estimated average amount of antimicrobials to which pigs within a batch were exposed during their lifetime using information about antimicrobial purchase, farm demographics, and pig movements from national registers.

Antimicrobial exposure was measured in animal defined doses per kilogram pig (ADD_kg_).

The ADD_kg_ is defined as the average approved dose for the main indication in the particular animal species for treatment of one kilogram pig. The ADD_kg_ can be used across age groups, as it is independent of animal bodyweight^42^. The ADD is equivalent to defined daily doses used in human medicine^43^. VetStat (the register on antimicrobial purchases) contains information about the number of ADD_kg_ that a package or vial of product contain. Combining this information with the number of packages purchased and the number of pigs makes it possible to calculate the ADD_kg_ per pig (equation (2)).

2 $${{\rm{A}}{\rm{D}}{\rm{D}}}_{{\rm{k}}{\rm{g}}}=\frac{{\rm{A}}{\rm{m}}{\rm{o}}{\rm{u}}{\rm{n}}{\rm{t}}\,{\rm{o}}{\rm{f}}\,\,{\rm{p}}{\rm{r}}{\rm{o}}{\rm{d}}{\rm{u}}{\rm{c}}{\rm{t}}\,{\rm{s}}{\rm{o}}{\rm{l}}{\rm{d}}\,{\rm{m}}{\rm{g}}/{\rm{k}}{\rm{g}}}{{{\rm{A}}{\rm{D}}{\rm{D}}}_{{\rm{k}}{\rm{g}}}/{\rm{m}}{\rm{g}}\ast {\rm{n}}{\rm{u}}{\rm{m}}{\rm{b}}{\rm{e}}{\rm{r}}\,{\rm{o}}{\rm{f}}\,{\rm{f}}{\rm{i}}{\rm{n}}{\rm{s}}{\rm{h}}{\rm{e}}{\rm{r}}{\rm{s}}\,{\rm{i}}{\rm{n}}\,{\rm{b}}{\rm{a}}{\rm{t}}{\rm{c}}{\rm{h}}}$$

Antimicrobial exposure was calculated per antimicrobial class and dispersing form. Antimicrobial agents were grouped following the classification structure provided by VetStat.

The use of antimicrobials in the Danish pig population is heterogeneous, with many farms using little or none of a specific antimicrobial class. Therefore, antimicrobial exposure (measured in ADD_kg_) was categorised according to the quartiles for the total exposure of antimicrobials for each of the rearing periods: piglet (birth−7 kg), weaner (7 kg–30 kg), finisher (30 kg–slaughter), and aggregated lifetime exposure (the sum of exposure in each period). The following categories were used:

1. No exposure (a value of zero)
2. Very low exposure (>0–25^th^ percentile)
3. Low exposure (>25^th^ percentile - Median)
4. High exposure (>Median −75^th^ percentile)
5. Very High exposure (>75^th^ percentile).

### Other variables

The number of slaughter pigs present at the farm was calculated based on data in national registers. Farm size was added to the analyses using a calculated number of finishers present at the farm. The number of age groups (sows with piglets, weaners, and finishers) present at the farm was also included in the analyses.

The number of farms at which the pigs in the batch had been in was calculated by tracing the pigs back in time using pig movement data. The variable was categorised as one, two, or more than two.

### Statistical analyses

All statistical analyses were carried out in R^44^ using RStudio^45^.

Multivariable logistic regression analyses were performed for *ermB* (low/high level), *sulI* (absence/presence), and *sulII* (absence/presence). In addition, multivariable linear regression analyses were performed for *ermF*, *tet*(M), *tet*(O), and *tet*(W), using log-transformed RQ levels to improve the homogeneity of variance and normality of residuals. Both the logistic and linear regression analyses were carried out in four steps. Backwards elimination was performed at each step, starting with the variables with the highest p-value and using a Bonferroni corrected significance level of 0.05 divided by the number of variables in the model for the β-estimates to eliminate the non-significant variables. The Bonferroni corrected p-value was used to correct for multiple comparisons. Furthermore, an ANOVA was used to test the overall effect of the final variables, and all non-significant variables were excluded, again using a Bonferroni correction. Antimicrobial classes already included in the model at the previous step were not included in the next step. The following variables were included in the models:

1. Categorical explanatory variables: categorised antimicrobial exposure variables for the piglet, weaning and finishing periods for both group and individual treatment for the 11 classes of antimicrobials, the number of farms from which the pigs originated, and the categories for *ermB*, *sulI*, and *sulII*.
  - Continuous explanatory variables: the number of slaughter pigs, the number of other age groups present at the farms, and the log transformed RQ values of *ermF*, *tet*(M), *tet*(O), and *tet*(W).
2. The total amount of antimicrobials for the piglet, weaning, and finishing periods.
3. The total amount of each antimicrobial class used over the lifetime of the pigs for both individual and group treatment.
4. The total amount used per antimicrobial class over the lifetime of the pigs.

Some farms had non-detects for *ermF* and *tet*(M), which were included in the regression analyses as ‘NA’.

To assess multicollinearity, we calculated Spearman’s correlation coefficients among all continuous explanatory variables, using ρ > 0.8 as a cut-off.

The adjusted R^2^ for the linear regression analyses of the final model was used to estimate the percentage of variation in the genes explained by the model. For the logistic regression analyses, McFadden’s pseudo R^2^ (calculated using the pR2 function in the pscl package^46^) was used as an equivalent.

Pairwise significant differences among individual levels of significant categorical variables were assessed using the LS-means package^47^ for the variables in the final models.

### Data availability

The data generated and analysed in the current study are not publicly available due to the agreement for obtaining data. Prior to collection of the data a written agreement was signed ensuring that no other than the project group (named persons) could obtain AMR data for individual farms, and subsequently follow the batches. However, summarised data are available from the corresponding author on reasonable request.

## Electronic supplementary material

Supplementary Information
