# Supplementary material for: Association between selected antimicrobial resistance genes and antimicrobial exposure in Danish pig farms
Source: Sci Rep. 2017 Aug 29;7:9683. doi: 10.1038/s41598-017-10092-9 (PMC5575052; doi:10.1038/s41598-017-10092-9)
Supplement: Supplementary file 1 — Supplementary Information [file 41598_2017_10092_MOESM1_ESM.pdf]

Title page

Title: Association between selected antimicrobial resistance genes and antimicrobial exposure in Danish pig farms

Authors and affiliations

Anna Camilla Birkegård*, Tariq Halasa, Kaare Græsbøll, Julie Clasen, Anders Folkesson, Nils Toft

Division for Diagnostics & Scientific Advice, National Veterinary Institute, Technical University of Denmark, Kemitorvet, Building 204, 2800 Kgs. Lyngby

*corresponding author, acbir@vet.dtu.dk

Supplementary Tables showing the results of the final regression model

Table S1: Estimates of regression coefficient (β), Standard Error (SE), Odds Ratio (OR) together with the Confidence Interval (CI) and the p-value of the final multivariable logistic regression model of the association between antimicrobial resistance (AMR) genes, antimicrobial exposure, and other factors for having high levels of *ermB* compared to low levels

Category of explanatory variable	Explanatory variable	Level[+]	β-estimate	SE	OR	CI	p-value[†]
	Intercept	-	-0.015	0.82	-	-	-
AMR genes	*ermF*, log(RQ)*	-	0.35	0.11	1.42	[1.15;1.76]	0.001
Antimicrobial exposure, weaning period	Macrolides, group	No[b,d]	Ref	-	-	-	<0.0001
		Very low[a]	0.98	0.30	2.65	[1.48;4.80]	0.001
		Low	0.91	0.43	2.48	[1.05;5.68]	0.03
		High[a]	1.37	0.42	3.95	[1.73;8.88]	0.001
	Tetracycline, group	No	Ref	-	-	-	0.004
		Very low	-0.08	0.30	0.93	[0.51;1.68]	0.8
		Low[e]	0.52	0.37	1.69	[0.81;3.48]	0.2
		High	-0.48	0.61	0.62	[0.17;1.89]	0.4
		Very High[c]	-2.22	0.80	0.11	[0.02;0.47]	0.006
Antimicrobial exposure, finishing period	Macrolides, group	No[c,d]	Ref	-	-	-	<0.0001
		Low[a,d]	2.49	0.34	12.05	[6.26;23.93]	<0.0001
		High[a,c]	4.19	0.47	66.06	[28.11;181.55]	<0.0001
	Tetracycline, group	No[d]	Ref	-	-	-	0.002
		Very low[d]	0.44	0.42	1.55	[0.67;3.48]	0.3
		Low	-0.26	0.37	0.77	[0.36;1.55]	0.5
		High[a,b]	-1,88	0.63	0.15	[0.04;0.47]	0.003

SE: Standard error, OR: Odds ratio, CI: Confidence interval of odds ratio.
†The p-value for the reference level is an overall test of significance with a Bonferroni correction to adjust for multiple comparisons.
+For categorical explanatory variables there are different levels. There are for the antimicrobial exposure levels up to five levels of exposure (see Fig. 1 for number of levels per antimicrobial exposure variable). Results from the LS-means analysis showed that the effect was significantly different from: a no exposure, b very low exposure, c low exposure, d high exposure, and e very high exposure.
*Continuous variable on log scale.
Ref: Reference level.
Group and individual refer to how the antimicrobial was administrated whereas the total refers to the total amount that the pigs have been exposed to.

Table S2: Estimates of regression coefficient (β), Standard Error (SE), statistic, and p-value of the final multivariable linear regression model of the association between of antimicrobial resistance (AMR) genes, antimicrobial exposure, and other factors on *ermF* levels measured as log(RQ-values)

Category of explanatory variable	Explanatory variable	Level[+]	β-estimate	SE	Statistic	p-value[†]
	Intercept	-	-9.40	0.52	-	-
AMR genes	*sulI*	absent[i]	Ref	-	-	<0.0001
		present[h]	0.30	0.09	3.16	0.002
	tet(M), log(RQ)*	-	-0.24	0.07	-3.71	0.0002
	tet(W), log(RQ)*	-	0.69	0.13	5,37	<0.0001
Antimicrobial exposure, weaning period	Lincomycin, group	No[b]	Ref	-	-	<0.0001
		Very low[a]	0.67	0.11	6.06	<0.0001
		Low	1.03	0.79	1.29	0.20
	Macrolides, group	No[b]	Ref	-	-	<0.0001
		Very low[a]	0.38	0.12	3.29	0.001
		Low	0.36	0.15	2.33	0.02
		High	0.02	0.17	0.10	0.92
Antimicrobial exposure, finishing period	Lincomycin, individual	No[b]	Ref	-	-	<0.0001
		Very low[a]	0.35	0.09	3.72	0.0002
	Macrolides, group	No[c,d]	Ref	-	-	<0.0001
		Low[a]	0.67	0.16	4.25	<0.0001
		High[a]	0.92	0.15	6.19	<0.0001
Other variables	Number of farms	1[m,n]	Ref	-	-	0.0005
		2[l]	0.62	0.14	4.30	<0.0001
		>2[l]	0.66	0.16	4.23	<0.0001

SE: Standard error

[†]The p-value for the reference level is an overall test of significance with a Bonferroni correction to adjust for multiple comparisons.

[+]For categorical explanatory variables there are different levels. The *ermB* gene is categorised as either high level or low level whereas the *sulI* gene is categorised as absent or present. The number of farms is referring to the number of farms that the pigs in the batch is originating from and is categorised into three groups: 1, 2 or more than 2 (2+). There are for the antimicrobial exposure levels up to five levels of exposure (see Fig. 1 for number of levels per antimicrobial exposure variable). Results from the LS-means analysis showed that the effect was significantly different from: [a] no exposure, [b] very low exposure, [c] low exposure, [f] low level of ermB, [g] high level of *ermB*, [h] absence of *sulI*, [i] presence of *sulI*, [l] 1 farm, [m] 2 farms, and [n] more than two farms.

*Continuous variable on log scale.

Ref: Reference level.

Group and individual refer to how the antimicrobial was administrated.

Ext.: extended.

Sim: simple.

Table S3: Estimates of regression coefficient (β), Standard Error (SE), Odds Ratio (OR) together with the Confidence Interval (CI) and the p-value of the final multivariable logistic regression model of the association between antimicrobial resistance (AMR) genes, antimicrobial exposure, and other factors for presence of *sulI* compared to absence

Category of explanatory variable	Explanatory variable	Level[+]	β-estimate	SE	OR	CI	p-value[†]
	Intercept	-	1.35	0.60	-	-	-
	*ermF** log(RQ)	-	0.24	0.07	1.28	[1.12;1.46]	<0.0001
	sulII	Absent[k]	Ref	-	-	-	<0.0001
		Present[j]	1.28	0.18	3.63	[2.54;5.24]	<0.0001
Antimicrobial exposure, piglet period	Ext. penicillin, total	No[b]	Ref	-	-	-	0.01
		Very low[a]	-0.79	0.26	0.46	[0.27;0.75]	0.004
	Tetracycline, total	No[b]	Ref	-	-	-	0.005
		Very low[a]	0.50	0.18	1.65	[1.16;2.35]	

SE: Standard error, OR: Odds ratio, CI: Confidence interval of odds ratio.
[†]The p-value for the reference level is an overall test of significance with a Bonferroni correction to adjust for multiple comparisons.
[+]For categorical explanatory variables there are different levels. The *ermB* gene is categorised as either high level or low level whereas the *sulII* gene is categorised as absent or present. There are for the antimicrobial exposure levels up to five levels of exposure (see Fig. 1 for number of levels per antimicrobial exposure variable). Results from the LS-means analysis showed that the effect was significantly different from: [a] no exposure, [b] very low exposure, [c] low exposure, [d] high exposure, [f] low level of ermB, [g] high level of *ermB*, [j] absence of *sulII*, and [k] presence of *sulII.*
*Continuous variable on log scale.
Ref: Reference level.
Individual refers to how the antimicrobial was administrated whereas the total refers to the total amount that the pigs have been exposed to.
Ext.: extended.

Table S4: Estimates of regression coefficient (β), Standard Error (SE), Odds Ratio (OR) together with the Confidence Interval (CI) and the p-value of the final multivariable logistic regression model of the association between antimicrobial resistance (AMR) genes, antimicrobial exposure, and other factors for presence of *sulII* compared to absence

Category of explanatory variable	Explanatory variable	Level[+]	β-estimate	SE	OR	CI	p-value[†]
	Intercept	-	-0.75	0.24	-	-	-
AMR genes	*sulI*	Absent[i]	Ref	-	-	-	<0.0001
		Present[h]	1.31	0.18	3.72	[2.62;5.32]	<0.0001
Antimicrobial exposure, weaning period	Aminoglycoside, individual	No[b]	Ref	-	-	-	0.0002
		Very low[a]	0.63	0.20	1.87	[1.28;2.76]	0.001
Other variable	Number of farms	1[n]	Ref	-	-	-	0.03
		2	0.53	0.25	1.71	[01.04;2.79]	0.03
		2+[l]	0.86	0.28	2.35	[1.37;4.05]	0.002

SE: Standard error, OR: Odds ratio, CI: Confidence interval of odds ratio.

[†]The p-value for the reference level is an overall test of significance with a Bonferroni correction to adjust for multiple comparisons.

[+] For categorical explanatory variables there are different levels. The *sulII* gene is categorised as absent or present. The number of farms is referring to the number of farms that the pigs in the batch is originating from and is categorised into three groups: 1, 2 or more than 2 (2+). There are for the antimicrobial exposure levels up to five levels of exposure (see Fig. 1 for number of levels per antimicrobial exposure variable). Results from the LS-means analysis showed that the effect was significantly different from: [a] no exposure, [b] very low exposure, [h] absence of *sulI*, [i] presence of *sulI*, [l] 1 farm, and [n] more than two farms

*Continuous variable on log scale.

Ref: Reference level.

Group and individual refer to how the antimicrobial was administrated.

Table S5: Estimates of regression coefficient (β), Standard Error (SE), statistic, and p-value of the final multivariable linear regression model of the association between of antimicrobial resistance (AMR) genes, antimicrobial exposure, and other factors on *tet*(M) levels measured as log(RQ-values)

Category of explanatory variable	Explanatory variable	Level[+]	β-estimate	SE	Statistic	p-value
	Intercept	-	-7.54	0.19	-	-
AMR genes	*ermF*, log(RQ)*	-	-0.09	0.02	-4.10	<0.0001
	tet(W) , log(RQ)*	-	0.41	0.08	5.25	<0.0001
Antimicrobial exposure, lifetime	Ext. penicillin, individual	No[b]	Ref	-	-	0.004
		Very low[a]	-0.24	0.06	-3.69	0.0002
	Pleuromutilin, individual1	No[b]	Ref	-	-	0.0002
		Very low[a]	0.24	0.06	3.69	0.0002
Other variables	Other age groups [‡]		-0.15	0.04	-3.71	0.0002

SE: Standard error

[†]The p-value for the reference level is an overall test of significance with a Bonferroni correction to adjust for multiple comparisons.

[+]For categorical explanatory variables there are different levels. There are for the antimicrobial exposure levels up to five levels of exposure (see Fig. 1 for number of levels per antimicrobial exposure variable). Results from the LS-means analysis showed that the effect was significantly different from: [a] no exposure and [b] very low exposure.

*Continuous variable on log scale.

‡Continuous variable. Other age groups refer to the number of other age groups that are present at the farm.

Ref: Reference level.

Individual refers to how the antimicrobial was administrated whereas the total refers to the total amount that the pigs have been exposed to.

Ext.: extended.

Table S6: Estimates of regression coefficient (β), Standard Error (SE), statistic, and p-value of the final multivariable linear regression model of the association between of antimicrobial resistance (AMR) genes, antimicrobial exposure, and other factors on *tet*(O) levels measured as log(RQ-values)

Category of explanatory variable	Explanatory variable	Level[+]	β-estimate	SE	Statistic	p-value[†]
	Intercept	-	-2.10	0.05	-	-
AMR genes	*tet*(W), log(RQ)*	-	0.34	0.05	10.45	<0.0001
Antimicrobial exposure, weaning period	Tetracycline, individual	No[b]	Ref	-	-	0.009
		Very low[a]	-0.07	0.03	-2.60	0.009
Other variable	Number of slaughter pigs per 1,000 pigs[‡]	-	0.04	0.009	4.28	<0.0001

SE: Standard error

[†]The p-value for the reference level is an overall test of significance with a Bonferroni correction to adjust for multiple comparisons.

[+]For categorical explanatory variables there are different levels. There are for the antimicrobial exposure levels up to five levels of exposure (see Fig. 1 for number of levels per antimicrobial exposure variable). Results from the LS-means analysis showed that the effect was significantly different from: [a] no exposure, [b] very low exposure, [c] low exposure, and [d] high exposure.

*Continuous variable on log scale.

‡Continuous variable.

Ref: Reference level.

Individual refers to how the antimicrobial was administrated whereas the total refers to the total amount that the pigs have been exposed to.

Ext.: extended.

Sim: simple.

Table S7: Estimates of regression coefficient (β), Standard Error (SE), statistic, and p-value of the final multivariable linear regression model of the association between of antimicrobial resistance (AMR) genes, antimicrobial exposure, and other factors on *tet*(W) levels measured as log(RQ-values)

Category of explanatory variable	Explanatory variable	Level[+]	β-estimate	SE	Statistic	p-value[†]
	Intercept	-	0.15	0.19	-	-
AMR genes	*ermF*, log(RQ)*	-	0.05	0.01	4.69	<0.0001
	sulI	Absent[i]	Ref	-	-	0.004
		Present[h]	0.09	0.03	3.36	0.0008
	tet(M), log(RQ)*	-	0.08	0.02	4.73	<0.0001
	tet(O), log(RQ)*	-	0.28	0.04	7.26	<0.0001
Antimicrobial exposure, weaning period	Sim. penicillin, individual	No[b]	Ref	-	-	0.0001
		Very low[a]	0.08	0.03	3.17	0.002
	Tetracycline, group	No[e]	Ref	-	-	<0.0001
		Very low	0.08	0.03	2.72	0.007
		Low	0.10	0.04	2.48	0.01
		High	0.13	0.06	2.38	0.02
		Very high[a]	0.23	0.05	4.26	<0.0001
Antimicrobial exposure, finishing period	Tetracycline, group	No[c,d]	Ref	-	-	<0.0001
		Very low[d]	0.13	0.05	2.68	0.008
		low[a,d]	0.20	0.04	5.47	<0.0001
		High[a,b,c]	0.32	0.04	7.78	<0.0001

SE: Standard error
[†]The p-value for the reference level is an overall test of significance with a Bonferroni correction to adjust for multiple comparisons.
[+]For categorical explanatory variables there are different levels. The *sulI* gene is categorised as absent or present. There are for the antimicrobial exposure levels up to five levels of exposure (see Fig. 1 for number of levels per antimicrobial exposure variable). Results from the LS-means analysis showed that the effect was significantly different from: [a] no exposure, [b] very low exposure, [c] low exposure, [d] high exposure, [e] very high exposure, [h] absence of *sulI*, and [i] presence of *sulI*.
*Continuous variable on log scale.
Ref: Reference level.
Group and individual refer to how the antimicrobial was administrated.
Ext.: extended.
Sim: simple.
